# Single-molecule analysis reveals that DNA replication dynamics vary across the course of schizogony in the malaria parasite *Plasmodium falciparum*

**DOI:** 10.1038/s41598-017-04407-z

**Published:** 2017-06-21

**Authors:** Slavica Stanojcic, Nada Kuk, Imran Ullah, Yvon Sterkers, Catherine J. Merrick

**Affiliations:** 1 University of Montpellier, Faculty of Medicine, Laboratory of Parasitology-Mycology, Montpellier, F34090 France; 2 Centre for Applied Entomology and Parasitology, Faculty of Natural Sciences, Keele University, Keele, Staffordshire, ST55BG UK; 3 CNRS 5290 - IRD 224 - University of Montpellier (UMR “MiVEGEC”), Montpellier, F34090 France; 4 University Hospital Centre (CHU), Department of Parasitology-Mycology, Montpellier, F34090 France

## Abstract

The mechanics of DNA replication and cell cycling are well-characterized in model organisms, but less is known about these basic aspects of cell biology in early-diverging Apicomplexan parasites, which do not divide by canonical binary fission but undergo unconventional cycles. Schizogony in the malaria parasite, *Plasmodium*, generates ~16–24 new nuclei via independent, asynchronous rounds of genome replication prior to cytokinesis and little is known about the control of DNA replication that facilitates this. We have characterised replication dynamics in *P*. *falciparum* throughout schizogony, using DNA fibre labelling and combing to visualise replication forks at a single-molecule level. We show that origins are very closely spaced in *Plasmodium* compared to most model systems, and that replication dynamics vary across the course of schizogony, from faster synthesis rates and more widely-spaced origins through to slower synthesis rates and closer-spaced origins. This is the opposite of the pattern usually seen across S-phase in human cells, when a single genome is replicated. Replication forks also appear to stall at an unusually high rate throughout schizogony. Our work explores *Plasmodium* DNA replication in unprecedented detail and opens up tremendous scope for analysing cell cycle dynamics and developing interventions targetting this unique aspect of malaria biology.

## Introduction

The malaria parasite *Plasmodium* belongs to an unusual lineage of protozoans, the Apicomplexa, which are characterised by very divergent cell cycles^1^. The *Plasmodium* cell cycle varies at different stages of the parasite’s lifecycle, as the parasite circulates between mosquito and human hosts, but at no stage does its cell cycle resemble that of any model system such as yeast cells, which divide by binary fission. In contrast, *Plasmodium* divides primarily by schizogony: a unique syncytial process that is also inadequately modelled by syncytial replication events such as those of early insect embryos^2^, where many nuclei within the same cytoplasm replicate synchronously, controlled by waves of diffusible regulators. Schizogony, by contrast, can involve asynchronous replication of nuclei within the same cytoplasm, and may be somewhat more analogous to the replication of certain hyphal fungi^3^.

A *Plasmodium* schizont produces many nuclei (not necessarily 2^n^) via multiple rounds of genome replication inside a single cell, prior to a final cytokinesis event in which the new nuclei are divided up into daughter merozoites which then burst out of the host cell and invade new cells^4^. The process of schizogony occurs entirely inside the confines of a host cell: either an erythrocyte in blood-stage schizogony or a hepatocyte in hepatic schizogony. Thus, the replication of these obligate intracellular stages is constrained by the size and intracellular environment of the host cell: a particular limitation in the erythrocyte, which is an anucleate cell of just ~10 μm diameter. All the symptoms of malaria in humans are caused by blood-stage parasites. In the most lethal human malaria species, *P*. *falciparum*, blood-stage schizogony takes ~48 hours and produces ~16–24 merozoites per cell.

The *Plasmodium* cell cycle is a phenomenon of fundamental biological interest due to its many unusual features. It is also of considerable clinical interest, because the parasitaemia achieved in human infections can vary greatly in different human hosts and a high parasitaemia is still one of the better clinical predictors of severe malaria^5, 6^. Parasitaemia is essentially determined by the balance between parasite growth rate and immune clearance, therefore parasite growth rate can have a direct influence on the development of clinical disease. Indeed, even when placed in *in vitro* culture, the growth of different *P*. *falciparum* strains varies – both in terms of cell cycle period and in terms of the number of merozoites produced per schizont^7^. What regulates this strain-to-strain variation remains unknown.

The *Plasmodium* cell cycle has until recently been relatively understudied, even in the blood-stage which is the most accessible for *in vitro* experiments, although certain key advances have recently been made^8, 9^. Cell cycle studies in this organism have proved difficult partly because schizogony does not obviously follow the canonical G1, S, G2 and M phases defined in binary fission^4, 10, 11^, and partly because many of the tools used in model cells are not easily transferable to *Plasmodium* ^12–14^. For example, synchronising agents such as aphidicolin and hydroxyurea do not block the cell cycle as tightly as they do in human cells, flow cytometry as it is used in human cells to measure DNA content from 1n to 2n is complicated by the large and variable number of genomes that are made per schizont, and labelling agents to identify actively-replicating DNA have not been usable in *Plasmodium*. In view of this, we recently developed a method for labelling nascent DNA replication in *Plasmodium* with modified nucleosides, which was not previously possible because the biochemistry of this parasite prevented it from incorporating exogenously-added thymidine analogues^15^. Our method allows the dynamics of *Plasmodium* DNA replication to be investigated with unprecedented spatial and temporal resolution. Here, the method is exploited to measure the dynamics of replication across the course of blood-stage schizogony at the single-molecule level, using molecular combing of DNA fibres and immunofluorescent detection of thymidine analogues incorporated into nascently-replicated DNA. Molecular combing is a very powerful technique that is frequently used in DNA replication and genome stability studies^16, 17^, as it provides a unique way to monitor the activation of replication origins and the progression of replication forks at the single molecule level. The technique involves the linearisation and uniform stretching (at 2 kb/μm) of single DNA fibres on glass slides after the successive incorporation of two modified nucleosides into newly-synthesized DNA. Importantly, this allows the accurate measurement of inter-origin distances (IODs), as well as the symmetry and velocity of progressing replication forks^18^. Such information has never previously been obtainable from malaria parasites, nor to our knowledge from any other species with such a highly A/T-biased genome or such an unusual cell cycle.

## Results

### Development of DNA fibre labelling in blood-stage *P. falciparum* parasites

**Figure 1:**
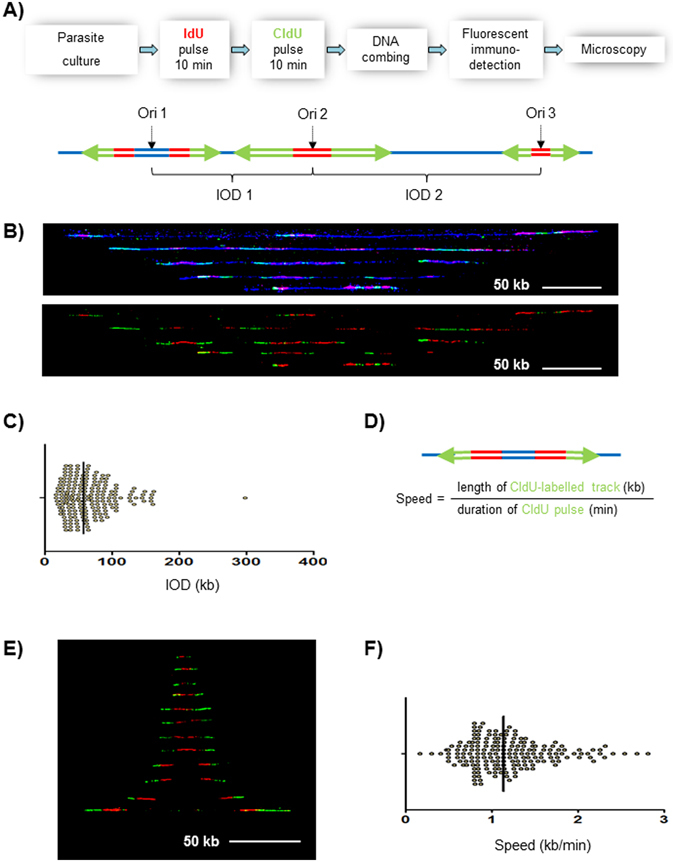
DNA fibre labelling to measure replication fork rates and inter-origin spacing in blood-stage *P. falciparum* parasites. (**A**) Upper panel: Outline of the experimental approach. Blood-stage *P*. *falciparum* parasites were successively labelled with 10-minute pulses of IdU and CldU, then chromosomal DNA was combed on silanized coverslips. DNA (blue) and modified nucleotides (IdU, red; CldU, green) were immuno-detected. Lower panel: Schematic showing some of the signal patterns observed on labelled DNA fibres. The presumed positions of the replication origins are indicated in the middle between bidirectional replication forks (Ori 1–3). The inter-origin distance (IOD) was measured as centre to centre distance between two adjacent progressing bidirectional forks. Green arrows represent the direction of the replication forks’ progression. (**B**) Representative DNA fibres from mixed-stage parasites. Upper panel: DNA fibres are in blue, IdU in red and CldU in green; lower panel show the IdU and CldU tracks extracted from the upper panel. 50 kb scale bars are indicated. (**C**) Dot plot showing distribution of inter-origin distances (IODs) measured in asynchronous blood stage forms of *P*. *falciparum*. Black bar represents the median value. (**D**) Schematic showing bidirectional replication forks. Replication fork velocity was calculated by dividing the green CldU track length by the duration of the second, CldU, pulse. (**E**) Bidirectional replication forks taken from different microscopic fields were artificially assembled and centred to the position of the presumed origins. Red tracks, IdU; green tracks, CldU. For clarity, counterstaining of DNA is not shown. Scale bar, 50 kb. (**F**) Dot plot showing distribution of replication fork speeds measured in asynchronous blood stage forms of *P*. *falciparum*. Black bar represents the median value.

DNA fibre labelling and molecular combing methodology was adapted to blood-stage *P*. *falciparum* parasites to permit single-molecule analysis of replication dynamics. Firstly, it was necessary in all experiments to use parasites that express a viral thymidine kinase (TK) and are able to convert modified nucleosides into nucleotides and incorporate them into nascent DNA^15^. Secondly, it was necessary to remove the haemozoin from labelled parasites prior to molecular combing of their genomes, since haemozoin crystals prevented the efficient combing of DNA fibres. Thirdly, the methodology for labelling nascent DNA replication in *P*. *falciparum* had previously been developed using only bromodeoxyuridine (BrdU)^15^, whereas in this work it was necessary to use iodo- and chlorodeoxyuridine (IdU and CldU) because two sequential and distinguishable pulse-labels were required to give directionality to the labelled tracks that represent active replication forks^19, 20^. The very different sizes of the two halogen substituents on IdU and CldU are distinguishable with two different antibodies, whereas BrdU combined with either IdU or CldU cannot be distinguished^21, 22^. The effects of IdU and CldU on the growth of *P*. *falciparum* were therefore checked because this parasite is unusually sensitive to BrdU toxicity – although, importantly, there is no evidence for any acute toxic effect during the short period of pulse-labelling used in these assays – and all halogen groups were found to have very similar effects (Fig. S1). Fourthly, the close spacing of replication origins discovered in these cells necessitated very short-duration pulse-labelling in order to label discrete tracts of nascent DNA before they merged together. 10-minute pulses of IdU followed by 10 minutes of CldU were used (Fig. 1A,B); longer periods resulted in the majority of replication forks merging and consequently fork velocity could not be measured (Fig. S2).

DNA fibres were prepared using the above-described method, after pulse-labelling a mixed-stage culture of TK-expressing 3D7 parasites. Two parameters were then calculated: the spacing of replication origins (‘inter-origin distance’, or ‘IOD’) in kb and the velocity of replication forks in kb/min. IOD was calculated by measuring centre-to-centre distances between adjacent replication bubbles on the same fibre (Fig. 1A,C). Fork velocity was calculated by dividing the length of the second, CldU-labelled, track by the 10-minute duration of the CldU pulse-label (Fig. 1D): representative bidirectional replication forks used for this analysis are shown in Fig. 1E. Approximately 200 measurements were made for each parameter.

Replication forks moved at a mean rate of 1.19 kb/min (median 1.14 kb/min) (Fig. 1F) and the mean IOD was 65.0 kb (median 58.4 kb) (Fig. 1C). All replication parameters are tabulated in Table 1. Since the *P*. *falciparum* genome is ~23 Mb, an IOD of 65 kb would translate to an estimate of 354 active replication origins per genome (or 396 origins using the median IOD of 58.4 kb) (Table 1).

**Table 1:** DNA replication parameters (column statistics) in asynchronous blood-stage parasites and in synchronous parasites at Stages 1–3.

	asynchronous	synchronous stage 1	synchronous stage 2	synchronous stage 3
**IOD** (**kb**)				
Number of values	198	241	240	271
Minimum (kb)	16.09	18.01	19.48	9.38
25% Percentile (kb)	37.52	45.96	43.57	36.86
Median (kb)	58.36	68.52	59.08	53.17
75% Percentile (kb)	84.37	100.8	84.27	78.70
Maximum (kb)	297.7	326.7	231.7	169.1
Mean (kb)	65.00	78.83	68.08	60.62
**Velocity** (**kb/min**)				
Number of values	189	219	228	195
Minimum (kb/min)	0.1675	0.2345	0.2680	0.1208
25% Percentile (kb/min)	0.8375	1.005	0.8482	0.5360
Median (kb/min)	1.139	1.388	1.176	0.9715
75% Percentile (kb/min)	1.431	1.796	1.527	1.380
Maximum (kb/min)	2.815	4.650	4.189	3.063
Mean (kb/min)	1.194	1.461	1.249	1.023
**Asymmetrical forks** (**long fork to short fork ratio**)				
Number of values	57	98	81	92
Minimum (long/short fork ratio)	1	1	1	1
25% Percentile (long/short fork ratio)	1.003	1.014	1.005	1.003
Median (long/short fork ratio)	1.048	1.080	1.050	1.099
75% Percentile (long/short fork ratio)	1.186	1.304	1.220	1.308
Maximum (long/short fork ratio)	2.808	3.726	4.324	2.787
Mean (long/short fork ratio)	1.186	1.289	1.270	1.241
**Estimation of active origin number**				
23 Mb/median IOD (kb)	396	338	390	434
23 Mb/mean IOD (kb)	354	291	338	377
**Number of forks**				
symmetric forks		54	49	46
asymmetric forks		44	32	46
unidirectional forks		15	7	20

### Replication parameters vary across the course of schizogony

**Figure 2:**
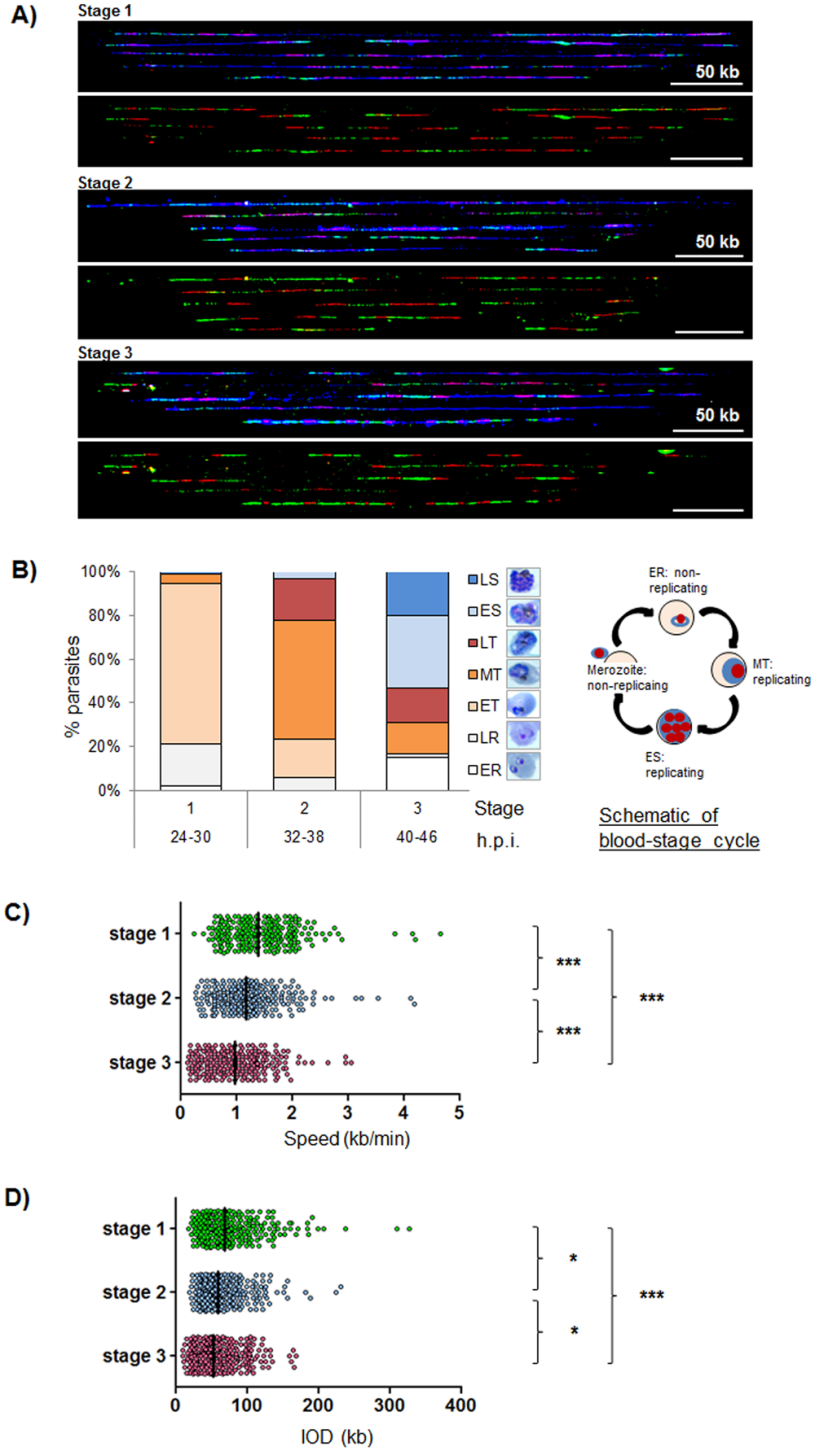
Replication parameters vary across the course of schizogony. (**A**) Representative DNA fibres from synchronous blood-stage parasites at Stages 1–3, labelled as in Fig. 1B. (**B**) Developmental stages of parasites harvested at Stages 1–3. Parasites were classified as: ER small rings, LR large rings, ET early trophozoites (less than half the width of host cell), MT middle trophozoites (more than half the width of, but not entirely filling, host cell), LT late trophozoite (parasite filling all or nearly all of host cell), ES early schizont (discrete nuclear masses visible within parasite), LS late schizont (defined merozoites visible). 100 parasites were counted at each timepoint. The adjacent schematic shows these stages within the erythrocytic replicative cycle. Stage 1 contains predominantly early trophozoites plus some late rings; Stage 2 contains predominantly mid-trophozoites with some early and late trophozoites; Stage 3 contains predominantly early schizonts with some late schizonts and late trophozoites. (**C** and **D**) Comparative analysis of replication fork speed (**C**) and inter-origin distances (**D**) in synchronous parasites. Black bars on dot plots indicate median values. The two-tailed Mann-Whitney test was used to calculate the corresponding P values (*P < 0.05; **P < 0.01; ***P < 0.001).

Over the course of S-phase in human cells, replication velocity tends to speed up and IODs become longer^23^. This reflects the replication dynamics of a single genome, whereas schizogony is fundamentally different, involving several repeated rounds of genome replication over ~20–24 hours within a single cell. Because replication in a schizont can occur asynchronously^4^, different nuclei within the same schizont could be starting or finishing genome replication at any time. However, this does not mean that replication parameters, averaged across all daughter nuclei, must necessarily be constant throughout schizogony. Within the cell, the metabolic and spatial environment changes greatly from an early trophozoite to a late schizont^4, 24^ and this could affect the progress of DNA replication globally. Therefore, replication parameters were measured in synchronised populations of parasites at the early, middle and late stages of the ~20-hour replicative portion of the 48-hour cell cycle, designated as Stages 1–3 (Fig. 2A,B). These stages correspond to very early trophozoites (plus ring-stage parasites that are not yet in S-phase, termed Stage 1), middle/late trophozoites (Stage 2), and primarily segmenting schizonts together with some remaining late trophozoites (Stage 3).

**Figure 3:**
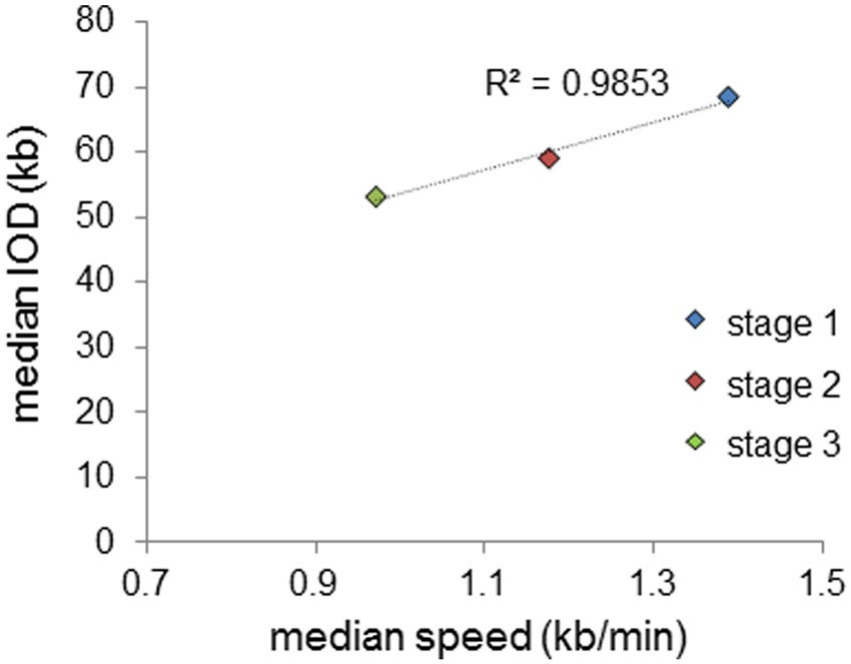
Inter-origin distances and fork velocities are positively correlated. Positive correlation between median inter-origin distances (IODs) and fork velocities in synchronous blood-stage parasites. Coefficient of determination (R^2^) is indicated.

Replication fork velocity was found to decrease by ~30% across the course of schizogony, from a mean of 1.46 kb/min in Stage 1 (early trophozoites) to 1.02 kb/min in Stage 3, a population dominated by schizonts (Fig. 2C, Table 1). Concomitantly, the IOD reduced from a mean of 78.8 kb to 60.6 kb (Fig. 2D, Table 1). Thus, the last round of nuclei to replicate within a schizont do so with different dynamics from the earliest round, and the estimated number of replication origins used per genome increases by ~30% (Table 1). The correlation between median IODs and fork velocities was analysed and the two were found to be highly positively correlated (coefficient of determination R^2^ = 0.9853, Fig. 3). Longer IODs observed in early trophozoites correlated with faster fork progression, while shorter IODs in predominantly schizont stages correlated with slower fork progression.

IOD measurements can potentially be biased by different DNA fibre lengths^25^, so the distribution of fibre lengths, as well as DNA counterstaining and the total number of measurements made, are crucial parameters to gain robust information about replication dynamics^25^. The lengths of combed DNA fibres were measured and compared in Stages 1–3 and also in the asynchronous population. In total, the DNA fibre lengths measured in all samples summed to more than three haploid genome sizes (Table S1). The distribution of DNA fibre lengths was very similar in all stages (Fig S3A and Table S1) and fibre lengths were not statistically different in any of the samples (Fig. S3B), confirming that our IOD comparisons are not biased by DNA fibre lengths.

**Figure 4:**
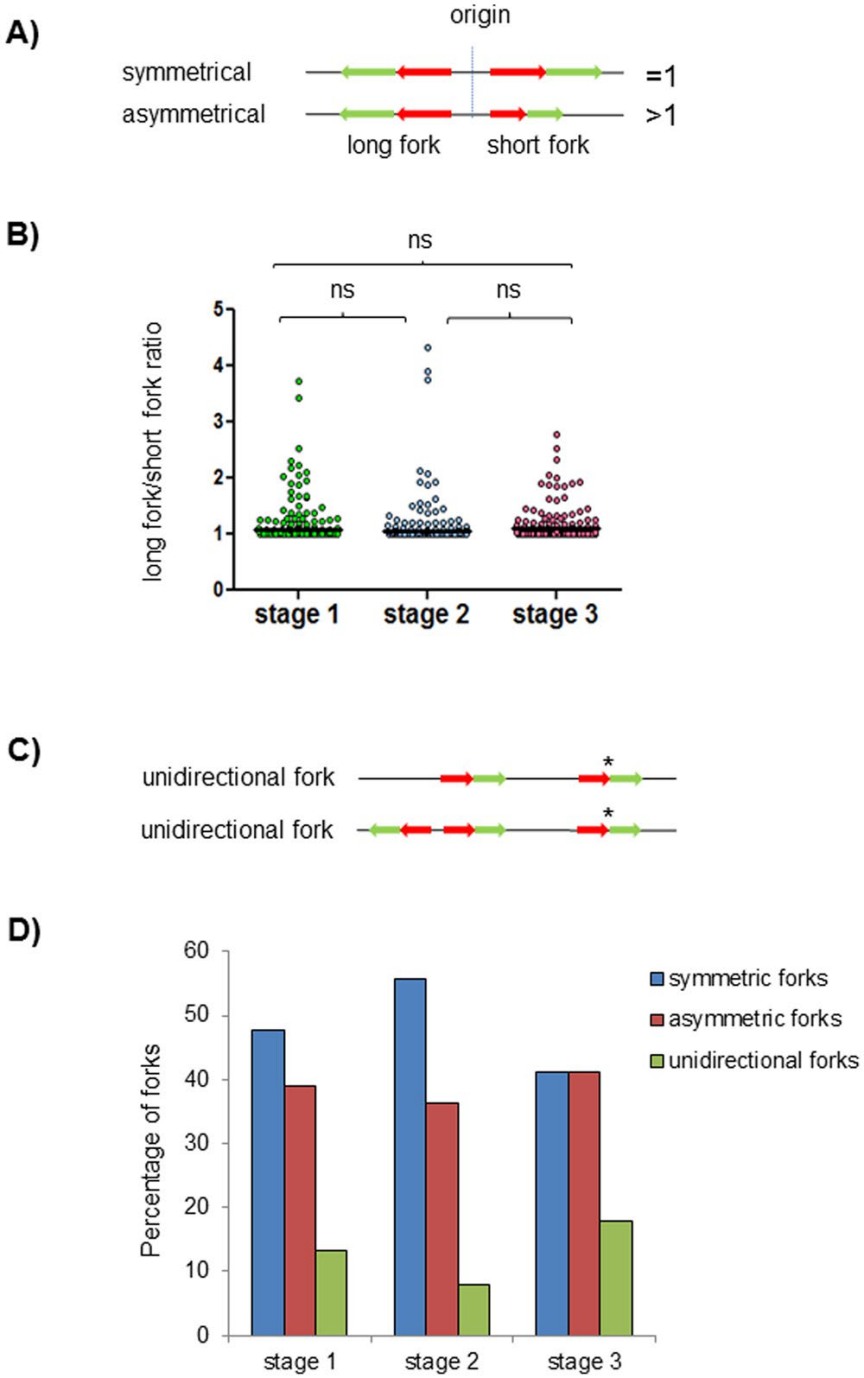
Asymmetric and unidirectional replication forks occur throughout schizogony. (**A**) Schematic representation of symmetrical and asymmetrical bidirectional forks. The long fork/short fork ratio corresponds to the ratio of the longer IdU + CldU track over the shorter IdU + CldU track. A ratio > 1 indicates fork asymmetry, while a ratio of 1 indicates symmetry. (**B**) Distribution of long fork/short fork ratios in synchronous parasites at Stages 1–3. Median values are indicated with black bars. P values were calculated using the two-tailed Mann-Whitney test, with significance set at 0.05. ns, not significant. (**C**) Schematic showing unidirectional replication forks. Only forks indicated with asterisks were counted as unidirectional. In the top fibre, the left fork was not considered as unidirectional because the fibre is finished and was probably broken between the bidirectional forks. (**D**) Percentage of symmetric, asymmetric and unidirectional forks counted in synchronous blood-stage parasites at Stages 1–3. The groups were not significantly different by Chi-square test (P = 0.192).

The symmetry of replication forks on either side of each replication bubble was also quantified, as shown in Fig. 4A. Asymmetric replication forks can indicate that the shorter fork has encountered an obstacle to replication during the pulse-labelling period, such as collisions with transcriptional machinery, DNA damage events requiring repair or bypass, DNA secondary structures such as hairpins and G-quadruplexes, or inhibitory chromatin structures. In *P*. *falciparum*, transcriptional activity in particular is known to vary dramatically across the course of the cell cycle, being low in ring-stages, increasing during the trophozoite stage and dropping again in late schizonts prior to reinvasion^26^, so the density of replication obstacles might be expected to vary in the course of schizogony. In fact, no significant difference was found in the level of fork symmetry across schizogony (Fig. 4B), and when the proportion of fork pairs that were symmetric, asymmetric or unidirectional (Fig. 4C) was counted at each stage, there was again no significant difference, although a trend was apparent towards Stage 2 parasites having more symmetric forks and fewer unidirectional forks than Stage 3 (Fig. 4D). Furthermore, unusually high levels of unidirectional forks were seen at all stages: 8–18% of all forks measured were unidirectional, compared with only 4% in mammalian cells^27^.

### The amount of actively-replicating DNA per cell increases over the course of schizogony

There are several possible reasons for the slowing down of replication velocity as schizogony proceeds, one of which could be that the supply of nucleotides or their precursors becomes increasingly rate-limiting. This would be expected if the synthesis of nucleotides does not keep pace with the amount of replicating DNA in the cell – which might be expected to increase logarithmically as the number of nuclei increases. Data gathered here were therefore used to address the assumption that the nature of schizogony entails a logarithmic increase in actively replicating DNA.

**Figure 5:**
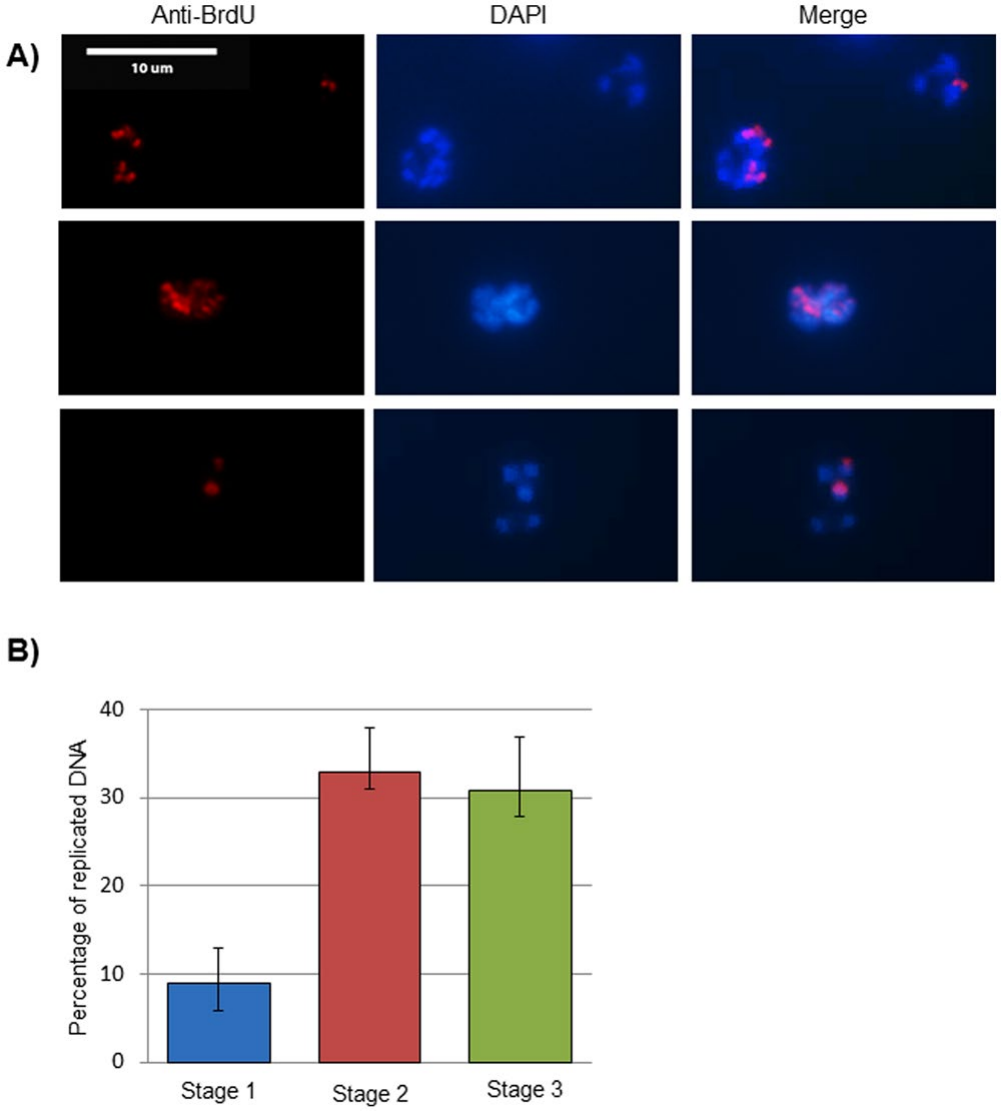
The amount of actively replicating DNA increases during schizogony. (**A**) Example immunofluorescence pictures of parasites exposed to 100 μM BrdU for 30 minutes, then labelled with DAPI (blue, all DNA) and an anti-BrdU antibody (red, newly-replicated DNA). (**B**) Percentage of actively replicating DNA during the 20-minute pulse-labelling period in synchronous blood-stage parasites at Stages 1–3. Mean percentages from three technical replicates are shown; in each replicate ~25 Mb of DNA fibres were analysed. Error bars show the range (minimum to maximum) across the three replicates. Images of combed DNA fibres were acquired randomly and the percentage of IdU + CldU tracks was calculated over the total DNA fibres length in all images (P = 0.0006 with one-way ANOVA analysis of variance; Stage 1 differs significantly from Stages 2 and 3 by T-test but Stages 2 and 3 do not differ).

Immunofluorescent imaging of whole parasites after a brief pulse-label with BrdU clearly shows that not all the nuclei in any parasite are replicating at any time, and indeed that replication sub-foci are discernible within individual nuclei (Fig. 5A). Therefore, to obtain a global measurement of how much DNA is replicating at Stages 1 to 3, the percentage of labelled DNA tracks over the total length of combed DNA fibres was calculated (Fig. 5B).

Only ~10% of fibres were replicating in Stage 1, while at Stages 2 and 3 the percentage stabilized at ~30%. This is to be expected because a quarter of the parasites in Stage 1 were still morphologically rings (Fig. 2B) and the rest, which appeared as early trophozoites, may or may not have actually commenced S-phase, whereas at Stages 2 and 3 the great majority of genomes should be in S-phase. (At Stage 3, ~10% ring-stage parasites were seen, having already undergone re-invasion, but since each ring has only 1n DNA content, versus ~16–24n from each schizont, only ~1% of the total fibres on these slides should come from non-replicating rings).

Setting aside the issue of asynchronous replication, it was assumed that a round of DNA replication takes approximately 4 hours, allowing 4–5 rounds of replication within the ~20-hour replicative phase and thus yielding the commonly observed number of ~16–24 merozoites per schizont. This estimate would hold true throughout the course of schizogony, because Fig. 3 shows that changes in fork velocity and IOD compensate for one another. Therefore, a Stage-2 parasite, at approximately 8 hours into S-phase, would contain ~2^2^n DNA content and a Stage-3 parasite, after a further 8 hours, ~2^4^n. Accordingly, since the percentage of actively replicating DNA does not change between these stages, parasites at Stage 3 will have 4-fold more actively replicating DNA than at Stage 2, and hence a 4-fold higher requirement for nucleotide precursors.

## Discussion

This work represents the first detailed investigation of DNA replication dynamics across the course of schizogony in blood-stage malaria parasites. The technique of DNA fibre labelling and molecular combing was successfully adapted for these parasites, permitting single-molecule analysis of replication. To achieve this, various modifications to the standard protocol were made because there are several challenges in using this technique with *P*. *falciparum*. Only genetically-modified TK-expressing lines can be used, and *P*. *falciparum* DNA fibres were found to be unusually brittle, possibly due to the exposure to alkaline buffers that was required to remove haemozoin, or simply to the high A/T content of this genome. Nevertheless, well-labelled and sufficiently long DNA fibres were obtained to permit meaningful analyses. This work is not only the first analysis of replication dynamics in any *Plasmodium* species; it is also the first, to our knowledge, performed in any genome with such a striking bias in base content, at > 80% A/T.

Replication parameters in *P*. *falciparum* were markedly different from those reported in most model systems. Mean IODs were 61–79 kb: about half the spacing reported for mammalian cells such as human primary keratinocytes (111 kb), mouse embryonic fibroblasts (136 kb), mouse embryonic stem cells (139 kb) and JEFF lymphoblastoid cells (147 kb)^25, 28, 29^. Closely-spaced replication origins are not, however, a particular feature of protozoan parasites because IODs at the opposite end of the spectrum have recently been measured in *Trypanosoma brucei* (160 kb) and *Leishmania mexicana* (226 kb)^27^. The IODs observed in *P*. *falciparum* were more similar to those in *Drosophila* Kc cells (mean IOD 73 kb^28^) and in budding yeast (mean IOD 46 kb^30^). Replication fork velocity in *P*. *falciparum* was slower than in most mammalian cells, averaging 1.0–1.5 kb/min compared with 1.4–1.9 kb/min in mouse embryonic stem cells, human primary keratinocytes, Chinese hamster embryonic fibroblasts and JEFF cells^25, 28, 29^. It was also much slower than the velocities measured in trypanosomatids (up to 2.6 kb/min in *L*. *mexicana* ^27^), but was faster than in *Drosophila* Kc cells (0.8 kb/min^28^).

The high density of active replication origins in *P*. *falciparum* might superficially suggest that schizogony could be completed much faster than it actually is. If an origin fired every 65 kb throughout the genome simultaneously then each round of replication could be completed within 27 minutes (65 kb divided by 1.19 kb/min, divided by two because replication is bidirectional), and thus five rounds could be completed in ~3 hours, yielding up to 32 merozoites. However, such calculations tend not to hold true in model systems because several factors act to reduce the theoretical speed. Firstly, origins do not all fire simultaneously. ‘Early’ and ‘late’ firing patterns, influenced by chromatin state, transcriptional activity, etc., occur in most eukaryotes^31^. Secondly, the spacing of replication origins has a wide distribution, ranging up to 327 kb at Stage 1 – a stretch of DNA that would take almost 2 hours to replicate if no additional origins were to fire. Thirdly, fork velocities are equally variable, with figures as low as 0.12 kb/min recorded at Stage 3. Fourthly, extra time may be required to resolve stalled replication forks at DNA damage events, fragile sites or transcriptional collisions. At all stages of schizogony many asynchronous and unidirectional forks were observed and these are often taken as evidence of impeded replication. In particular, the proportion of unidirectional forks was very high, at 8–18% compared to ~4% in mouse embryonic fibroblasts and ~2% in *Leishmania* parasites^27^. The appearance of unidirectional forks did not, however, vary significantly at different time points in schizogony, suggesting that transcriptional activity – which follows a well-characterised pattern in *P*. *falciparum* with the highest activity in the trophozoite stage^26^ – may not be the main impediment to replication forks. Instead, unconventional DNA structures may be responsible, since the *P*. *falciparum* genome has many low-complexity A/T tracts^32^ that tend to give rise to slipped-strand pairing and hairpins. It has been proposed that a similarly high proportion of unidirectional forks recently detected in *T*. *brucei* may be linked to the hairpin-forming potential of repetitive palindromes in the *T*.*brucei* genome^27^. Overall, a more realistic replication period per *P*. *falciparum* genome might be ~4 hours, allowing the observed average of 4–5 rounds of replication to occur within ~20–24 hours^4^.

In *P*. *falciparum*, replication velocity diminished across the course of schizogony: the opposite of the pattern normally seen in human cells^23^. One possible explanation would be a failure of nucleotide production to keep pace with the amount of replicating DNA, which is shown here to increase logarithmically during schizogony. This situation may be particular to the nature of schizogony (and other syncytial replication situations such as early embryos of *Drosophila* and *Xenopus*). By contrast, in human cells only one genome is replicated and a logarithmic increase in the amount of replicating DNA across S-phase is not expected to occur: instead, the increasing activity of ribonucleotide reductase^33, 34^ leads to increasing nucleotide pools throughout S-phase and replication velocity, which is directly influenced by nucleotide availability, increases accordingly^23, 35^. In *P*. *falciparum*, the derivation of the nucleotide pool inside the parasite is complex, with contributions from both *de novo* synthesis and salvage pathways^36, 37^. Detailed data on nucleotide pools across schizogony are not well established, but one metabolomic study which examined timepoints every 8 hours across the cell cycle suggests that there is not a logarithmic increase in most nucleotide precursors, which tend instead to follow a bell curve from 24 to 48 hours post-invasion^24^. We have previously shown that *P*. *falciparum* can pre-load its nucleotide pool with BrdU during the ring stage well before replication commences^15^, but nevertheless it may then fail to keep pace with the growing demand for nucleotides as schizogony proceeds.

Alternative explanations for reduced replication velocity are also possible. For example, the final round of replication could be more challenging than the first round if progressively more DNA damage occurs as the parasite’s metabolism proceeds. However, an increase in asymmetric forks was not observed over the course of schizogony, so this explanation is not supported by our data. The chromatin state may also change as the schizont becomes more crowded: indeed, a global change of chromatin structure has been reported during the *P*. *falciparum* blood-stage cell cycle^38, 39^. Nucleosome packaging decreases after invasion, reaching a minimum at the early trophozoite stage and proceeding to an intense packaging at the late schizont stage as merozoites become ready for the next invasion. This broadly reflects the changes in replication dynamics observed here, so it may be that replication speed is restricted by progressively compacting chromatin. Conversely, however, replisome impediments can favour the formation of heterochromatin in various different model systems (reviewed in ref. 40), so replication fork slowing may actually promote the formation of heterochromatin across the course of schizogony. Importantly, none of these explanations are mutually exclusive and all of them may contribute to the observed dynamics.

Whatever the explanation, it is apparent that parasites can progressively compensate for diminishing replication speeds by employing more closely-spaced origins as replication proceeds. This implies that the availability of ORC proteins and their ability to bind to the genome are not limiting factors, and indeed most transcriptomic analyses do show ORC transcription increasing throughout schizogony^41^. Secondly, this finding implies either that origin specification is very plastic and that it differs at Stage 1 versus Stage 3, or, if the same origins are laid down at every round of replication, that many more of these origins remain dormant at Stage 1 than at Stage 3. Plasticity of origin usage is common in eukaryotic cells, with many potential origins being licenced during G1 and only a small percentage being used during S phase (reviewed in ref. 42). Dormant origins can, however, be activated when replication forks are inhibited, allowing the timely completion of the replication programme^42–44^. Furthermore, our data imply that there is an intrinsic and global mechanism for balancing origin firing with replication velocity in *P*. *falciparum*. A similar correlation between fork velocity and IOD was previously described for lymphoblastoid cells, fibroblasts and trypanosomatids^27, 45^, suggesting that fork velocity and IOD are commonly co-regulated. Indeed, in Chinese hamster cells it has been demonstrated that a slowing of replication forks can trigger the recruitment of latent origins within minutes, allowing the completion of S-phase in the same overall time period^44^. In this system, the replication fork speed determines the spacing of anchorage regions of chromatin loops and this, in turn, controls the pattern of origin activation^44^. It seems that a mechanism for controlling plasticity of replication origin usage is universal and conserved throughout evolution.

The factors determining replication origin specification in *P*. *falciparum* remain unknown. In most model systems, with the exception of *S*. *cerevisiae*, there is no ORC-binding DNA sequence but rather a set of consensus features such as epigenetic marks and guanine quadruplexes that promote origin specification^46–48^. *P*. *falciparum* uses most of the canonical epigenetic histone marks^49^, some of which could contribute to origin specification, whereas guanine quadruplexes are an unlikely player because they are predicted to be much too rare, with only ~80 occurring outside the telomeres in this highly A/T-rich genome^50, 51^. However, poly-A/T tracts are contrastingly common^32^ and these are involved in origin specification in fission yeast^52^ and in *Xenopus* ^53^. Indeed, these would be common enough to facilitate plastic and variable origin specification in different rounds of replication as schizogony proceeds.

Finally, the key issue of how different nuclei can replicate asynchronously inside a single schizont while avoiding re-replication and the attendant risk of aberrant mitosis and aneuploidy still remains a mystery. The S-phase parameters established here, together with the prospects for using nascent DNA labelling at a whole-cell as well as a single-molecule level, have now laid vital groundwork for addressing this mystery. Several other fascinating cell-biological questions can also be addressed: how the asynchronous replication that occurs in schizogony is organised, monitored and controlled, how replication origins are specified in *P*. *falciparum*, and whether origin specification changes from the beginning to the end of schizogony.

## Materials and Methods

### Parasite culture

*P*. *falciparum* parasites of the 3D7 strain, genetically modified to express thymidine kinase^15^, were cultured as previously described^54^. Synchronised cultures were obtained by double treatment with 5% D-sorbitol^55^ 19 hours apart, leaving a 4–5 hour window of surviving ring-stages parasites. In the subsequent cycle, parasites were harvested at three stages of the replicative cycle, with parasite staging assessed at each stage via morphology using blood smears stained with the Hemacolor staining kit (Merck Millipore). At least 100 parasites were classified for their developmental stage at each timepoint.

### Preparation of agarose plugs for combing of *P. falciparum* DNA

Two modified nucleosides were used to label parasites for DNA molecular combing: iodo-deoxyuridine (IdU, Sigma) and chloro-deoxyuridine (CldU, Sigma), both dissolved in water and stored as frozen stocks at −20 °C prior to dilution into culture medium. Parasites were sequentially labelled for 10 minutes with 25 μM IdU, then for 10 minutes with 200 μM CldU, added directly to the culture without intermediate washing. After labelling, the cultures were immediately placed on ice to stop DNA replication. Parasites were removed from cells by addition of saponin to 0.1% final concentration, collected by centrifugation and washed three times in ice-cold PBS. They were resuspended at 1 × 10^8^ parasites per 100 μl of PBS containing 1% low-melting-point agarose (Dutscher), in order to embed the cells in agarose plugs. Plugs were incubated in 1 mL per plug of proteinase K buffer (10 mM Tris-Cl, pH 7.0, 100 mM EDTA, 1% N-lauryl-sarcosyl and 2 mg/mL proteinase K, pH 9.8) at 45 °C for 2 days with fresh solution added on the second day. Haemozoin is soluble at alkaline pH^56^ and it was therefore necessary to soak in alkaline buffer in order to remove the haemozoin prior to combing.

### DNA molecular combing

The complete removal of digested proteins and other degradation products from agarose plugs was performed by washing the plugs several times in TE50 buffer (50 mM EDTA, 10 mM Tris-Cl, pH 7.0). Protein-free DNA plugs were then stored in TE50 buffer at 4 °C or used immediately for combing. Agarose plugs were stained with YOYO-1 fluorescent dye (Molecular Probes) in TE50 buffer for 1 h, washed several times with TE50 buffer, resuspended in 100 μl of TE50 buffer and melted at 65 °C for 15 minutes. The solution was maintained at 42 °C for 15 minutes and then treated overnight with β-agarase (Sigma Aldrich). After digestion, 4 mL of 50 mM MES (2-(N-morpholino) ethanesulfonic acid, pH 5.7) was added very gently to the DNA solution and then DNA fibres were combed and regularly stretched (2 kb/μm) on silanized coverslips as described previously^16^.

Combed DNA was fixed for at least 2 h at 65 °C, denatured in 1 N NaOH for 20 minutes and washed several times in 1 × PBS. After denaturation, silanized coverslips bearing the DNA fibres were blocked with 1% BSA and 0.1% Triton X-100 in PBS. Immuno-detection was done with antibodies diluted in 1 × PBS, 0.1% Triton X-100, 1% BSA and incubated at 37 °C in a humid chamber for 60 min. Each step of incubation with antibodies was followed by extensive washes with 1 × PBS. Immuno-detection was done with anti-ssDNA antibody (1/100 dilution, Chemicon), mouse anti-BrdU antibody (1/20 dilution, clone B44 from Becton Dickinson) and rat anti-BrdU antibody (1/20 dilution, clone BU1/75 (ICR1) from Sera Lab), which recognize IdU and CldU respectively. The mouse anti-BrdU, clone B44, is derived from hybridization of mouse Sp2/0-Ag14 myeloma cells with spleen cells from BALB/c mice immunized with iodouridine-conjugated ovalbumin. It reacts with iodouridine and BrdU^21, 22^. The rat anti-BrdU antibody, clone BU1/75 (ICR1), cross reacts with chlorodeoxyuridine (CldU) but does not cross react with thymidine or iododeoxyuridine^21^. The secondary antibodies were goat anti-rat antibody coupled to Alexa 488 (1/50 dilution, Molecular Probes), goat anti-mouse IgG1 coupled to Alexa 546 (1/50 dilution, Molecular Probes), and goat anti-mouse IgG2a coupled to Alexa 647 (1/100 dilution, Molecular Probes). Coverslips were mounted with 20 μl of Prolong Gold Antifade (Molecular Probes) and dried at room temperature for 12 h.

Image acquisition was via a fully motorized Leica DM6000 microscope equipped with a CoolSNAP HQ2 1 CCD camera and controlled by MetaMorph (Roper Scientific). Images were acquired with a 40 × objective, where 1 pixel corresponds to 335 bp. Thus, at a magnification of 40 × , one microscope field of view corresponds to ~450 kb. Observation of longer DNA fibres therefore required the capture and assembly of adjacent fields. Inter-origin distances and the velocity of replication forks were measured manually using the MetaMorph software. Statistical analyses of inter-origin distances and velocities of replication forks were performed using Prism 5.0 (GraphPad). Two independent parasite cell-cycle timecourses, labelling and combing experiments were performed and the data from one representative experiment are presented here. Data from the second replicate experiment appear in Figure S4 and Table S2.

### Statistical analysis of DNA fibre data

Replication fork velocity was estimated on individual forks displaying an IdU track flanked by a CldU track. Only intact forks were analysed, as ascertained by DNA counterstaining. Fork asymmetry was calculated as the ratio of the longer track over the shorter track in pairs of progressing divergent forks. A longer fork/shorter fork ratio > 1 indicates asymmetry. Inter-origin distances were measured as the distance (in kb, from the stretching factor of 2 kb/μm) between the centres of two adjacent progressing forks located on the same DNA fibre. GraphPad Prism (GraphPad Software) was used to generate graphs and perform statistical analysis. DNA replication parameters generally do not display a Gaussian distribution^57^. Statistical comparisons of the distributions were therefore carried out using the nonparametric Mann–Whitney two-tailed tests that do not assume Gaussian distribution. The numbers of symmetric, asymmetric and unidirectional forks in the different cell types were compared using Chi-square tests. Statistical significance was set at p ≤ 0.05. The correlation between fork speed and IODs was analysed using linear regression and the coefficient of determination R^2^.

### Whole-cell Immunofluorescence

Parasites were pulse-labelled with 100 μM bromo-deoxyuridine (BrdU, Sigma) for 30 minutes, then prepared for immunofluorescence as air-dried blood smears. Smears were fixed for 5 mins in 4% formaldehyde/PBS, then for 2 mins in 50/50 methanol/acetone, then washed 3 times in PBS and blocked for 30 mins in 1% BSA/PBS. Slides were treated for 2 h with mouse monoclonal anti-BrdU antibody BU-1 plus nuclease (Amersham, kit RPN202), washed three times in 1% BSA/PBS, treated for 2 h with anti-mouse Cy3-conjugated secondary antibody (Stratech Scientific) diluted 1:5000 in BSA/PBS, then washed again three times with BSA/PBS. 0.2 μg ml^−1^ 4′,6-diamidino-2-phenylindole (DAPI) was included in the second wash. Slides were mounted in ProLong Diamond (Thermo Fischer Scientific) and examined using an EVOS FL imaging system (Fisher).

### Malaria SYBR Green I Fluorescence (MSF) assay

MSF assays for parasite growth were carried out essentially as previously described^15, 58^, plating synchronised trophozoite-stage parasites at 0.5% parasitaemia and 2% haematocrit in a 96-well format containing serial dilutions of modified nucleosides and then incubating for 48 h. To read these assays, the resultant cultures were mixed with MSF lysis buffer (Smilkstein *et al*., 2004) and SYBR Green I fluorescence was measured using the GloMax multidetection system (Promega). Percentage parasite growth was calculated relative to the fluorescence readouts from the control culture, incubated without added nucleosides (100% growth) and the fluorescence readouts from cultures treated with the maximum concentration of nucleosides (0% growth). All assays were carried out in triplicate. GraphPad Prism v.6.0 (GraphPad software Inc.) was used to plot growth inhibition curves via non-linear regression fitting, and thus to calculate 50% inhibitory concentration (IC50) values.

## Electronic supplementary material

Supplementary Information
