# Supplementary material for: Single-molecule analysis reveals that DNA replication dynamics vary across the course of schizogony in the malaria parasite Plasmodium falciparum
Source: Sci Rep. 2017 Jun 21;7:4003. doi: 10.1038/s41598-017-04407-z (PMC5479783; doi:10.1038/s41598-017-04407-z)
Supplement: Supplementary file 1 — Supplementary Information [file 41598_2017_4407_MOESM1_ESM.pdf]

Supplementary Information:

Single-molecule analysis reveals that DNA replication dynamics vary across the course of schizogony in the malaria parasite *Plasmodium falciparum*

Authors

Slavica Stanojcic[1], Nada Kuk[1], Imran Ullah[2], Yvon Sterkers[1,3,4 ¶*], Catherine J. Merrick[2 ¶*]

Affiliations & Contact Information:

[1] University of Montpellier, Faculty of Medicine, Laboratory of Parasitology-Mycology, Montpellier, F34090, France;

[2]Centre for Applied Entomology and Parasitology, Faculty of Natural Sciences, Keele University, Keele, Staffordshire, ST55BG, UK;

[3]CNRS 5290 - IRD 224 - University of Montpellier (UMR "MiVEGEC"), Montpellier, F34090, France;

[4]University Hospital Centre (CHU), Department of Parasitology-Mycology, Montpellier, F34090, France.

¶ These authors contributed equally to this work.

* To whom correspondence should be addressed.

yvon.sterkers@univ-montp1.fr (YS)

c.merrick@keele.ac.uk (CJM)

Figure S1

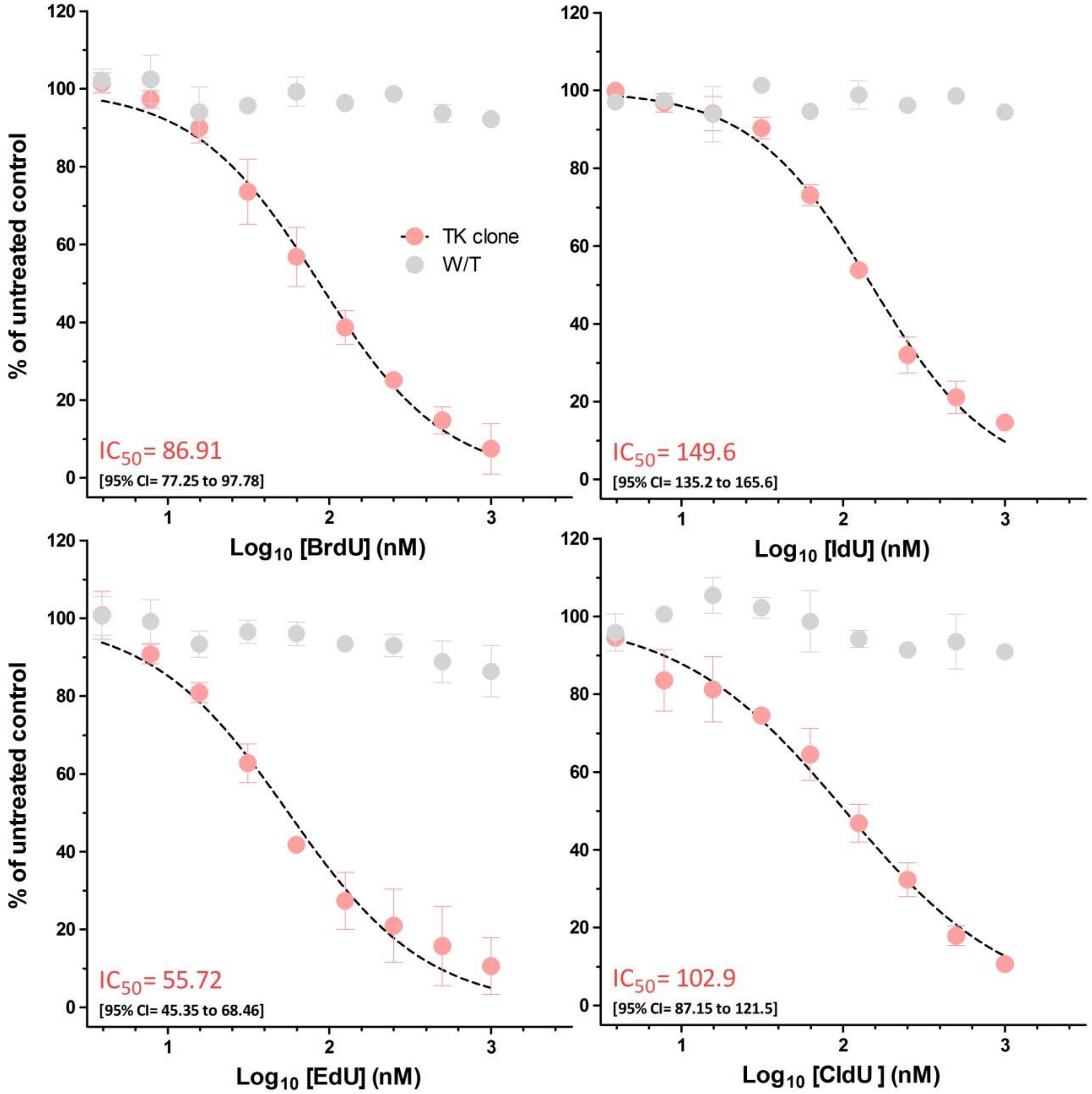

Figure S1:
Results of MSF assays on parasites treated for 48 h with a range of concentrations of CldU, BrdU, IdU and EdU. It was previously found that, although blood-stage parasites can progress normally through S-phase despite many hours of BrdU exposure, the exposed parasites then die in the late schizont stage (13). Therefore, the relative effects of CldU, BrdU and IdU on the growth of *P. falciparum* parasites were measured. All substituents were tested in parallel via a standard 48-hour parasite growth assay, trophozoites to trophozoites. Pink circles show TK-expressing parasites; wildtype (W/T) parasites that do not incorporate modified nucleotides, and are therefore insensitive to their toxic effect, are shown in grey for comparison. Error bars show standard deviation of triplicate readings. Results show that all halogen groups had very similar effects, with the IC50 for IdU (150nM) being slightly higher than BrdU or CldU (87 and 103nM respectively). 5-ethynyl-2'-deoxyuridine (EdU) was also tested and this proved the most toxic of all (IC50 56nM) although the ethynyl group represents the smallest modification and is not halogenated. Therefore, large substituting groups cannot be responsible for the toxicity of modified nucleosides, and all modified nucleosides can be used in *P. falciparum* with the expectation of similar parameters concerning cellular toxicity.

Figure S2

DNA fibres from asynchronous cells: labelled 20m IdU, 20m CldU

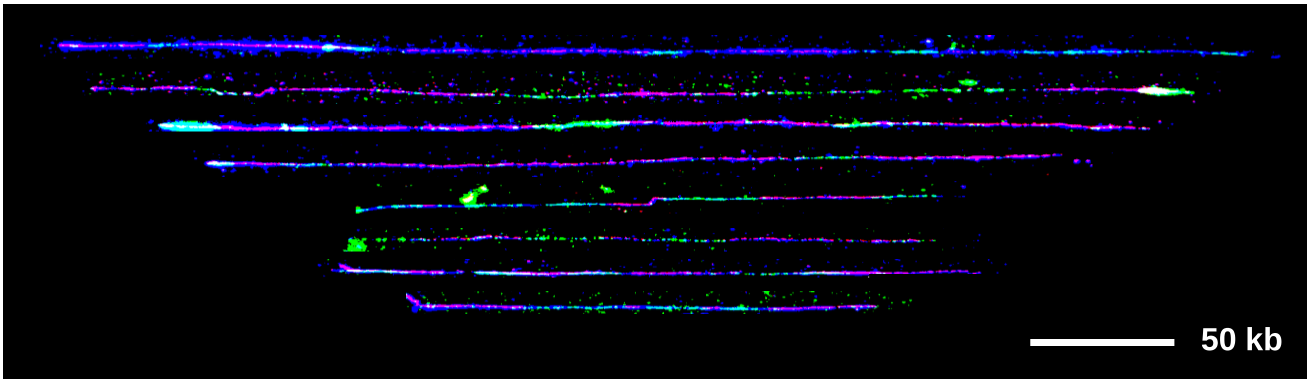

Figure S2:
Representative DNA fibres from asynchronous blood-stage parasites labelled for 20 min with IdU (red) and 20 min with CldU (green). DNA fibres are in blue. 50kb scale bar is indicated.

Figure S3

A)

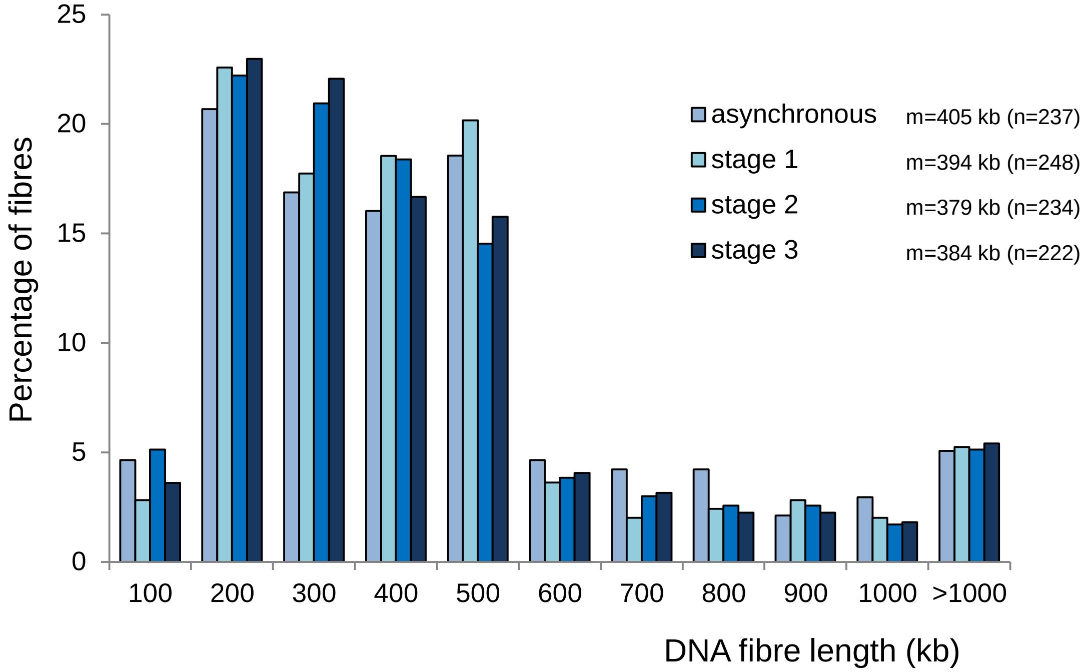

B)

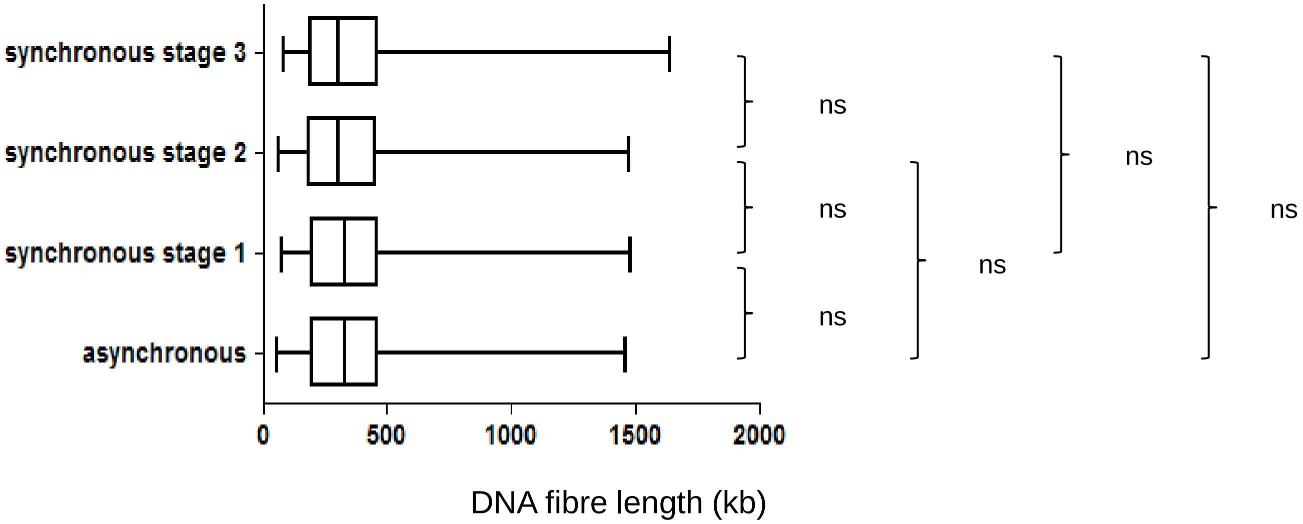

Figure S3:
A) Distribution of DNA fibre lengths, measured after combing on coverslips. The median fibre length in kb (m) and the number of measures (n) are indicated for each sample.
B) Comparative analysis of DNA fibre lengths. Boxes present 25–75% range with bars indicating medians. Whiskers present minimum–maximum range. The two-tailed Mann-Whitney test was used to calculate the corresponding P values.

Figure S4

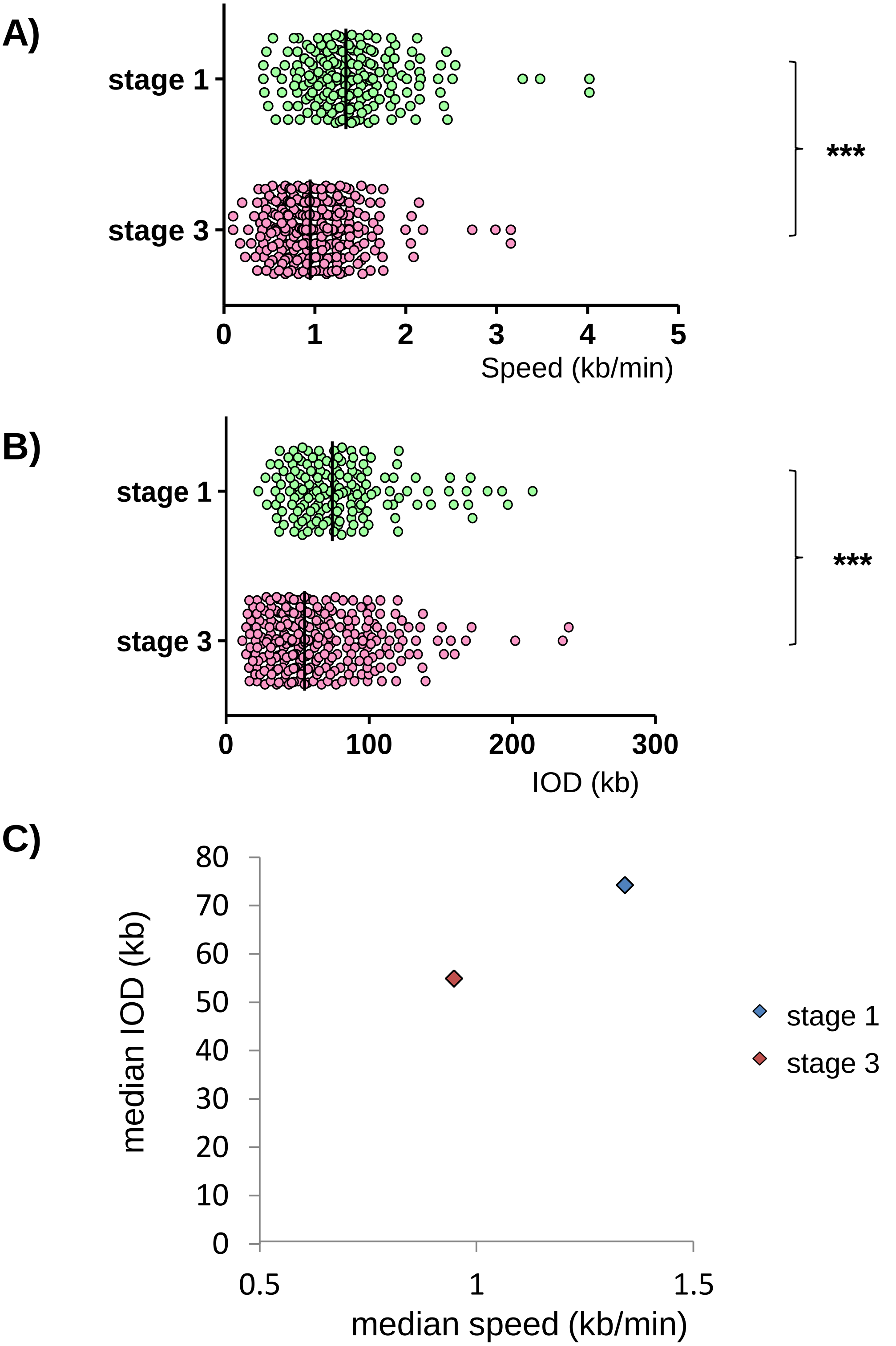

Figure S4: ***Replication parameters across the course of schizogony in a replicate experiment***

A, B) Comparative analysis of replication fork speed and inter-origin distances in synchronous parasites, showing the same trend as in main Figure 2C,D. Black bars on dot plots indicate median values. The two-tailed Mann-Whitney test was used to calculate the corresponding P values (***, $P < 0.001$).

B) Positive correlation between median inter-origin distances (IODs) and fork velocities in synchronous blood-stage parasites, as also shown in main Figure 3.

Table S1

	asynchronous stage	synchronous stage 1	synchronous stage 2	synchronous stage 3
Number of values	237	248	234	222
Minimum (kb)	57.33	76.39	60.43	83.23
25% Percentile (kb)	198.2	198.8	183.5	193
Median (kb)	331.6	332	302,8	305.1
75% Percentile (kb)	462.8	464.3	456.1	460.4
Maximum (kb)	1459	1473	1468	1637
Mean (kb)	405.1	393.9	378.6	384.3
Sum (kb)	96000	97694	88599	85314

Table S1:
Column statistics of DNA fibres' total length in asynchronous and synchronous parasites at Stages 1–3.

Table S2

	synchronous stage 1	synchronous stage 3
**IOD** (kb)		
Number of values (kb)	156	270
Minimum (kb)	22,60	11,39
25% Percentile (kb)	53,78	37,40
Median (kb)	74,25	54,91
75% Percentile (kb)	94,36	88,38
Maximum (kb)	214,3	239,5
Mean (kb)	80,03	65,35
**Speed** (kb/min)		
Number of values	188	298
Minimum (kb/min)	0,4355	0,1005
25% Percentile (kb/min)	1,072	0,7106
Median (kb/min)	1,342	0,9481
75% Percentile (kb/min)	1,643	1,242
Maximum (kb/min)	4,021	3,158
Mean (kb/min)	1,414	1,013

Table S2:
DNA replication parameters (column statistics) from the replicate experiment shown in Figure S4: synchronous blood-stage parasites at Stages 1 and 3. There is no statistically significant difference (Mann-Whitney test) between the parameters measured in the two replicate experiments.
